# Seasonal variation in BMI outcomes at 6 months: secondary analyses of a multidisciplinary healthy lifestyle programme for children and adolescents with obesity

**DOI:** 10.1007/s12519-025-01016-z

**Published:** 2026-02-06

**Authors:** José G. B. Derraik, Kima T. Costelloe, Cervantée E. K. Wild, Lisa E. Wynter, Mohammad Shahbaz, Paul L. Hofman, Yvonne C. Anderson

**Affiliations:** 1 Department of Paediatrics, Child and Youth Health, Faculty of Medical and Health Sciences, University of Auckland, Auckland, New Zealand; 2 Environmental–Occupational Health Sciences and Non-Communicable Diseases Research Centre, Research Institute for Health Sciences, Chiang Mai University, Chiang Mai, Thailand; 3 Department of Paediatrics, Health New Zealand | Te Whatu Ora Taranaki, New Plymouth, New Zealand; 4 Liggins Institute, University of Auckland, Auckland, New Zealand; 5 Curtin Medical School, Faculty of Health Sciences, Curtin University, Kent Street, Bentley, Perth, WA 6102 Australia; 6 The Kids Research Institute Australia, Perth, WA Australia; 7 Child and Adolescent Community Health, Child and Adolescent Health Service, Perth, WA Australia

**Keywords:** Lifestyle factors, Obesity, Pediatric, Random forest, Season

## Abstract

**Background:**

While international evidence suggests seasonal variations may influence outcomes of interventions for pediatric obesity, data for Aotearoa New Zealand are limited. We examined seasonal variations in changes in body mass index standard deviation score (BMI SDS) in young people with obesity enrolled in an intervention programme.

**Methods:**

We studied 397 children and adolescents (median = 10.1 years; range 3.7–16.8 years) from Whānau Pakari, a multidisciplinary community-based healthy lifestyle programme (initially a randomised clinical trial that subsequently transitioned into the regional childhood obesity service). Participants were stratified by season at entry and 6-month BMI SDS changes (Δ) were evaluated. Lifestyle factors were also assessed. Data were analysed using traditional linear models and machine learning (random forest).

**Results:**

68% of participants had BMI SDS reductions at 6 months (mean = − 0.16 SDS; *P* < 0.0001). Linear models showed seasonal variations in programme effectiveness, with BMI SDS reductions among summer (− 0.17 SDS), autumn (− 0.19 SDS) and winter (− 0.18 SDS) but not among spring entrants. Random forest modelling identified higher baseline BMI SDS and younger age as the most influential predictors of greater 6-month reductions in BMI SDS. Season of entry was more important than any single lifestyle factor; spring entrants exhibited attenuated reductions relative to other seasons.

**Conclusions:**

The season at programme entry was an important factor associated with intervention effectiveness. Spring entry was associated with attenuated BMI SDS reductions, likely due to the inclusion of the summer holidays within the 6-month intervention. These findings highlight the need for targeted support during such unstructured periods to improve participant outcomes.

**Graphical abstract:**

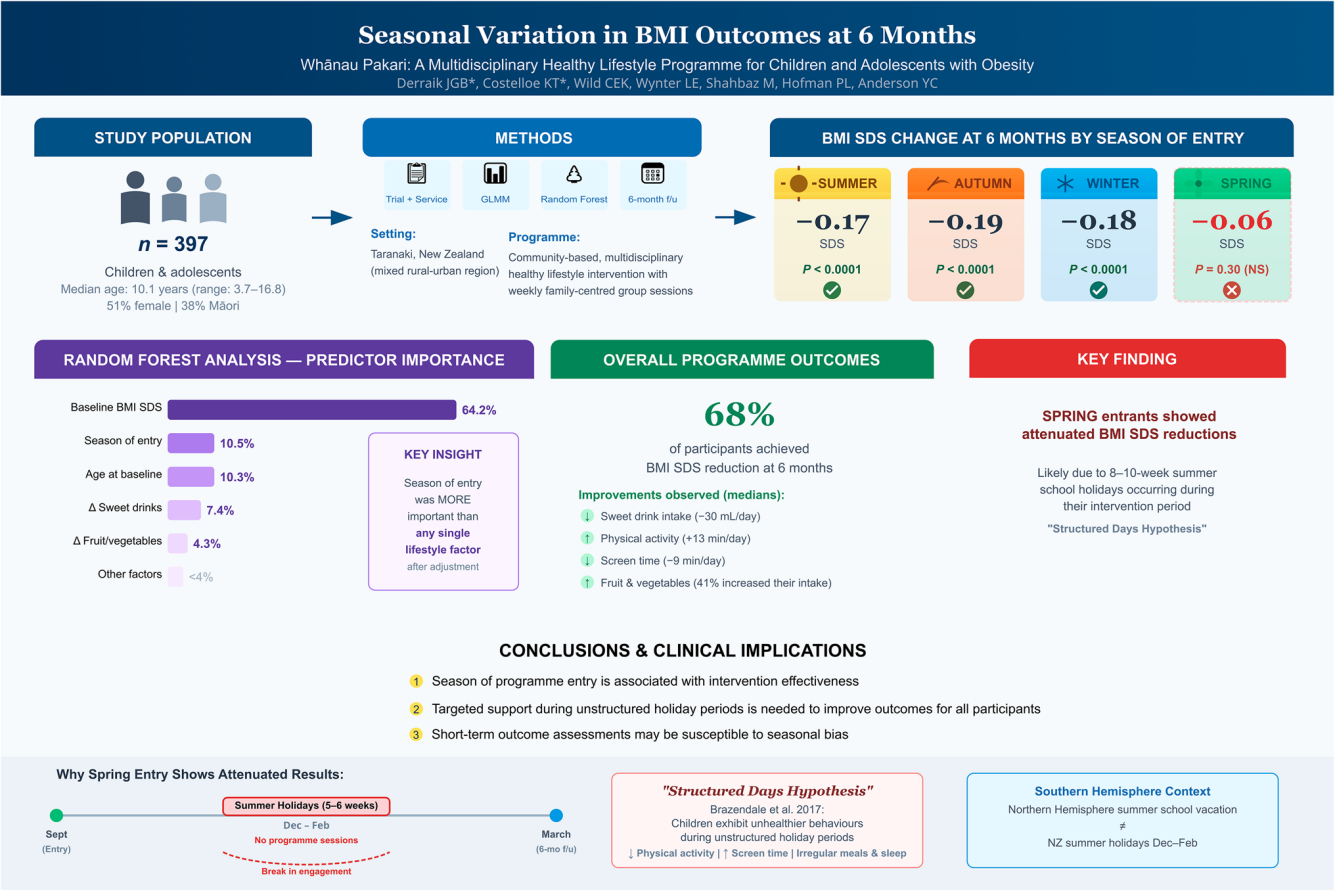

**Supplementary Information:**

The online version contains supplementary material available at 10.1007/s12519-025-01016-z.

## Introduction

Addressing childhood obesity remains a challenge in Aotearoa/New Zealand (henceforth referred to as NZ). Childhood obesity has a high prevalence in countries from the Organisation for Economic Co-operation and Development (OECD) [1]. In NZ, 12.5% of children aged 2–14 years are affected [2]. Overrepresentation is seen in those affected by socioeconomic deprivation, and Māori and Pacific children are also more likely to be affected [2]. Obesity can lead to weight-related health complications that result in greater morbidity and mortality [3].

Seasonal variations can influence adult obesity with evidence suggesting increased weight gain in winter. This may be due to lower physical activity levels and increased consumption of unhealthy foods [4, 5]. Conversely, among children in the Northern Hemisphere, greater weight gain was observed in summer [6–10]. This is likely associated with less routine in the summer holidays, increasing the likelihood of obesogenic behaviours such as reduced physical activity and increased dietary intake [11]. Brazendale et al. described this as the “Structured Days Hypothesis”, where children exhibit unhealthier behaviours during unstructured holidays and at weekends [11].

There are limited data on seasonal weight variations in NZ children and young people. In Australia, a small reduction in children's physical activity during the summer school term has been reported and attributed to higher temperatures [12]. However, the climate in Australia in most regions is markedly different from that in NZ. These distinct seasonal patterns (e.g., sunshine hours, temperature and rainfall) may affect behaviour (and thus weight gain). Nonetheless, seasonal sleep variations and physical activity patterns in NZ have been identified as potential protective factors against obesity [13–15].

Established in 2012, the Whānau Pakari programme is a community-based, multidisciplinary approach to addressing childhood obesity in Taranaki (a mixed rural–urban region of NZ) [16–18]. The programme was developed through community consultation and aims to improve healthcare equity, accessibility and cultural appropriateness for groups disproportionately affected by obesity [16–18]. This healthy lifestyle programme for children and young people involves demedicalised weight-related health assessments and healthy lifestyle education delivered through weekly family-centred group sessions [16–18]. Initially implemented as a randomised controlled trial (henceforth referred to as the “trial”), it transitioned into the regional childhood obesity service. The trial achieved high participation from Māori (47% compared to a population prevalence of 28%) [17, 18]. There were reductions in body mass index standard deviation score (BMI SDS) at 6 and 12 months in both high- and low-intensity intervention groups, which were not sustained at 24 months [17, 18]. The exceptions were participants in the high-intensity intervention with high attendance (≥ 70% of sessions) who maintained a reduction in BMI SDS of − 0.22 at 24 months [18]. The present study examined whether BMI SDS changes at 6 months were associated with the season of entry into the programme (trial or subsequent service) and also explored potential associations between season and underlying changes in lifestyle factors.

## Methods

### Programme and study design

As noted above, the Whānau Pakari programme evolved through two sequential phases. The initial trial (2012–2014) compared a high-intensity intervention (weekly group sessions during school term time for 12 months plus 6-monthly assessments) with a low-intensity control (6-monthly assessments and advice only) [16]. After the trial, the programme was adopted as the regional childhood obesity service in Taranaki (2014 onwards). The clinical service maintained core elements of the trial intervention while adjusting the weekly session duration to six months due to waning attendance in the second 6-month period. This was supported by evidence suggesting that ≥ 26 hours of contact time (equivalent to approximately 26 weekly one-hour sessions) is required to achieve meaningful BMI SDS reductions [19]. All participants received support from a multidisciplinary team, including a pediatrician, psychologist, dietitian, and physical activity coordinator, with screening and action for any identified weight-related comorbidities [16].

This study analysed combined data from both the trial and the subsequent clinical service to maximise statistical power and capture real-world data. The trial and service data could be integrated because intervention delivery and outcome measurements remained consistent across both phases during the first six months. This methodological consistency allowed for robust data pooling. Furthermore, referrals to the programme were accepted year-round which minimises the risk of seasonal bias. We are also unaware of other factors that may have biased the recruitment of participants into either the trial or the service. Our statistical analyses accounted for possible differential associations between study cohorts (i.e., trial or service).

### Participants

Eligibility criteria remained constant throughout both programme phases. Children and young people aged approximately 4.0 to < 18.0 years were eligible if their BMI was at or above the 98th percentile for age and sex, or at or above the 91st percentile with concurrent weight-related comorbidities, according to the UK 1990 reference data used for screening in NZ [20]. Participants also had a baseline assessment in January 2012–June 2020 and a follow-up assessment within 6 months ± 5 weeks (i.e., 183 ± 35 days). Exclusion criteria encompassed medical conditions affecting their ability to engage in physical activity, psychological conditions affecting group participation or the absence of committed family support [21].

### Data collection

Holistic clinical assessments occurred at the participant's home or a community venue agreed to by the family. Demographic information collected via caregiver questionnaires included the participant's age, biological sex (male or female) and self-reported ethnicity (based on prioritised ethnicity used by the health board at the time) [22]. Weight was measured using SECA 813 digital scales (SECA, Hamburg, Germany) and height with SECA 213 portable stadiometers. BMI was subsequently calculated. Waist circumference was also measured and the waist-to-height ratio was calculated. Participants were initially screened in clinic using the UK 1990 (UK90 or ‘UK Cole’) growth reference [20]. Weight, height and BMI were converted to age- and sex-specific standard deviation scores (SDS or *z*-scores) using the age-appropriate WHO growth system (Supplementary Methods; Supplementary Fig. 1).

Dietary patterns (24-h recall) were assessed using the modified Children’s Dietary Questionnaire (CDQ), adapted to the NZ context [23]. Physical activity levels were measured using the Children’s Physical Activity Questionnaire (C-PAQ) [24]. For children aged < 11 years, questionnaires were completed jointly by the caregiver and child. Caregivers reported the duration of sleep during the collection of medical history. Parameters of interest were self-reported (or proxy reported, where age-appropriate): physical activity levels, fruit and vegetable servings per day, time spent on screens, sleep duration and intake of sweet drinks.

### Statistical analyses

The primary outcome was change (Δ) in BMI SDS at 6 months. Secondary outcomes included the likelihood of a reduction in BMI SDS, changes in other anthropometric parameters and changes in diet and lifestyle measures. Participants were stratified by Southern Hemisphere season at baseline, defined by the meteorological criteria [25]: summer (December–February), autumn (March–May), winter (June–August) and spring (September–November). Our analytical approach combined traditional linear models with machine learning techniques.

#### Linear models

Demographic and anthropometric characteristics at baseline were summarised as means ± standard deviation (SD), medians [Q1, Q3] or *n* (%) and compared between seasons using Fisher’s exact tests for categorical variables, one-way ANOVA for continuous variables approximating a normal distribution on visual inspection, or non-parametric Kruskal–Wallis tests for skewed continuous variables.

Univariable (unadjusted) Δ from baseline (overall and within season) were examined using paired *t* tests, while between-season differences were assessed with one-way ANOVA. Differences were reported as mean Δ with 95% confidence intervals (CI). Linear associations with continuous predictors were analysed using Pearson’s or Spearman’s rank correlation coefficients.

Associations between season and Δ BMI SDS at 6 months were analysed using a generalised linear mixed model (GLMM). The model included family ID as a random intercept, accounting for the clustering of siblings, effectively nesting participant-level data within family clusters. Based on existing evidence of factors influencing childhood obesity intervention outcomes (particularly in NZ), the model included season (the primary exposure of interest), baseline BMI SDS, age at baseline, sex, ethnicity (Māori vs Non-Māori) and programme cohort (service vs trial) as fixed effects. We tested for a season*cohort interaction to assess for differential influences of the controlled trial environment and the routine service delivery on outcome; if non-significant (*P* ≥ 0.05), the interaction term was removed from the final model. This a priori variable selection approach avoided data-driven model building that could lead to overfitting with our sample size (*n* = 397). The same analytical approach was applied to other anthropometric outcomes (weight SDS, height SDS and waist-to-height ratio). GLMM results are presented as adjusted means (i.e., least-squares means) with 95% CI; between-group and within-group differences are reported as adjusted mean differences (aMD) with 95% CI.

Seasonal rates of BMI SDS reduction at 6 months were compared with a Fisher's exact test. Further, the likelihood of a BMI SDS reduction was assessed with a multivariable generalised linear model using a modified Poisson procedure with robust error variances [26]. This model included the same covariates as specified for the GLMM, with effect size expressed as the adjusted relative risk (aRR) with 95% CI.

Baseline dietary and lifestyle variables were compared between seasons using Kruskal–Wallis tests, followed by unadjusted pairwise comparisons using Wilcoxon rank-sum tests. Univariable overall and within-season Δ were assessed with Wilcoxon signed-rank tests. Multivariable ranked GLMMs were run to examine possible between-season Δ differences in these parameters compared to baseline and these were constructed as previously described. If there was evidence of between-season differences (*P* < 0.05 in the ranked GLMM), pairwise effect magnitudes were quantified using Wilcoxon-derived Hodges–Lehmann location shift estimates with 95% CI.

Data were analysed using SAS v9.4 (SAS Institute, Cary, NC, USA). All statistical tests were two-sided with significance set at *P* < 0.05. Given the exploratory nature of the study, planned pairwise comparisons between seasons were not adjusted for multiple comparisons to avoid inflating the risk of Type II errors, which could obscure clinically relevant patterns [27–29].

#### Random forest

Given the complex associations and potential non-linear relationships between lifestyle, demographic and clinical factors that may influence changes in BMI SDS, we used random forest modelling to capture non-linear effects and higher-order interactions that may not be readily detected by traditional linear models [30, 31]. Random forests can handle multiple correlated predictors and implicitly model interactions, making them suitable for identifying factors associated with Δ BMI SDS within our pediatric population.

Random forest analyses were conducted in *R* v4.4.1 [32] using the *ranger* package for model fitting, with supporting packages for data management and visualisation (*readxl*, *writexl*, *ggplot2*, *dplyr*, *caret*, *forcats*, *tibble* and *stringr*). Two random forest models were developed to predict Δ BMI SDS over the 6-month follow-up period. The base model incorporated demographic, clinical and temporal variables (season, programme cohort, sex, ethnicity, age at baseline and baseline BMI SDS). The expanded model included these variables plus dietary and lifestyle factors (i.e., sweet drink intake, physical activity levels, screen time, sleep duration and fruit/vegetable servings per day).

Missing values were imputed within each training fold [mean for continuous variables; mode (most frequent category) for categorical variables] ensuring no information from the corresponding test folds was used during model training. Model performance was evaluated using repeated fivefold cross-validation (10 repeats) to reduce variance and guard against overfitting.

Variable importance was assessed using permutation importance (mean increase in prediction error after permuting each predictor) as implemented in *ranger* [30]. Importance scores were averaged across cross-validation folds. For interpretability plots, we used out-of-fold (OOF) predictions, whereby each participant’s predicted outcome was generated exclusively from models that did not include that participant in training data.

## Results

### Study population

We studied 397 participants: 158 from the trial and 239 from the subsequent clinical service, including 30 families with siblings. Participants had a median age at baseline of 10.1 years (range 3.7–16.8 years) and a mean baseline BMI SDS of 3.29 (SD = 1.01); 51% were female (Table 1). At baseline, 69 participants were assessed in spring, 112 in summer, ﻿89 in autumn and 127 in winter (Table 1).

**Table 1:** Baseline demographics and anthropometric characteristics of study participants overall and according to the season at Whānau Pakari programme entry

Variables	Overall	Summer	Autumn	Winter	Spring	*P*
Demographics						
*n*	397	112	89	127	69	
Age, y	10.1 (7.0, 12.7)	10.3 (6.5, 12.9)	10.5 (7.9, 12.4)	9.6 (6.9, 12.0)	10.3 (7.2, 12.6)	0.83
Time from baseline, d	187 (178, 195)	184 (175, 192)	186 (179, 194)	187 (179, 196)	189 (175, 196)	0.50
Sex						
Females	204 (51%)	64 (57%)	40 (45%)	65 (51%)	35 (51%)	0.40
Males	193 (49%)	48 (43%)	49 (55%)	62 (49%)	34 (49%)	
Ethnicity^a^						
Māori	149 (38%)	43 (38%)	37 (42%)	46 (36%)	23 (33%)	0.74
Non-Māori	248 (62%)	69 (62%)	52 (58%)	81 (64%)	46 (67%)	
Programme						
Service	239 (60%)	69 (62%)	51 (57%)	57 (45%)	62 (90%)	< 0.0001
Trial	158 (40%)	43 (38%)	38 (43%)	70 (55%)	7 (10%)	
Anthropometry						
BMI SDS	3.29 ± 1.01	3.26 ± 0.95	3.32 ± 0.98	3.31 ± 0.98	3.24 ± 1.04	0.31
Weight SDS	2.97 ± 1.06	2.95 ± 0.99	3.09 ± 1.12	3.00 ± 1.02	2.80 ± 1.16	0.43
Height SDS	0.86 ± 1.02	0.82 ± 0.94	1.01 ± 1.00	0.94 ± 1.08	0.61 ± 1.03	0.10
WHtR	0.59 ± 0.05	0.58 ± 0.06	0.59 ± 0.05	0.58 ± 0.05	0.59 ± 0.06	0.31

Categorical data are *n* (%). For continuous variables, age and time data are the medians (lower quartile, upper quartile), with the remaining variables reported as the mean ± standard deviation. *P* values were derived from one-way ANOVA or Fisher’s exact tests, as appropriate, and refer to an overall difference between the seasons

*BMI* body mass index, *SDS* standard deviation score, *WHtR* waist-to-height ratio. Seasons were defined as per meteorological criteria [25]

^a^Based on self-prioritised ethnicity

Demographic and anthropometric characteristics at baseline were largely similar across the seasons at entry, except for a lower proportion of trial participants among spring entrants (Table 1). Importantly, the distribution of participants according to age and BMI SDS at baseline was also similar (Supplementary Fig. 2).

### Primary outcome: Δ BMI SDS

**Fig. 1:**
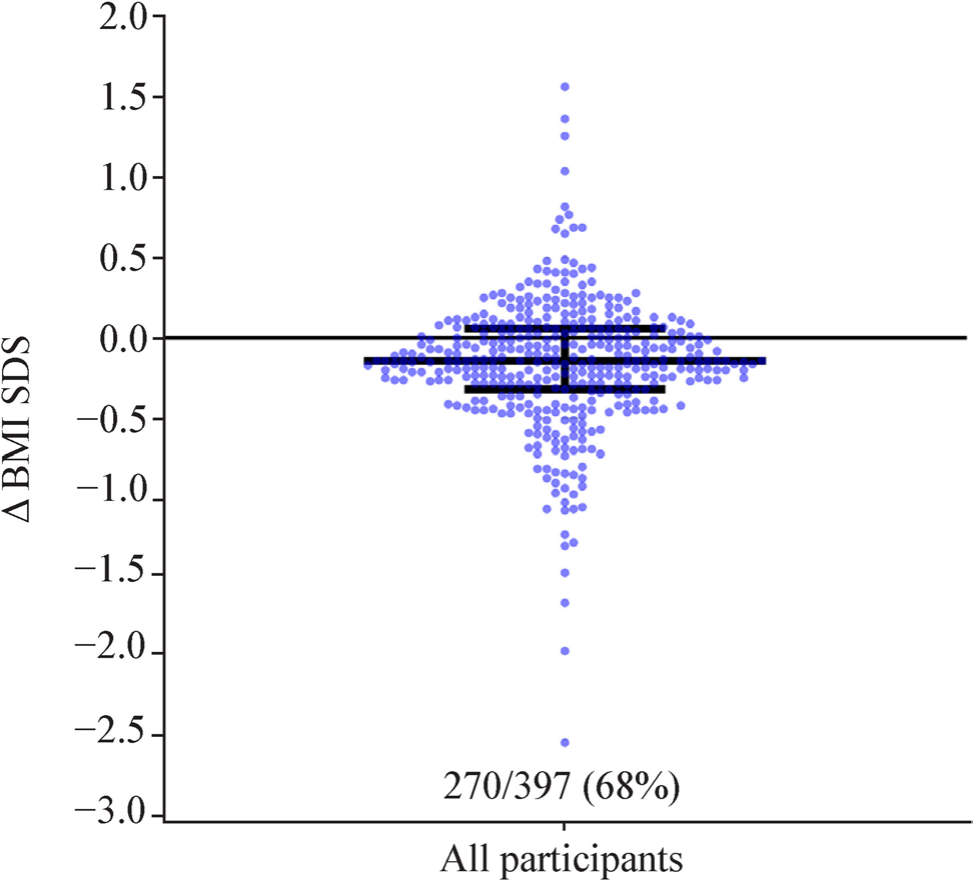
Sinaplot showing the distribution of changes (Δ) in body mass index standard deviation scores (BMI SDS) at 6 months compared to baseline among study participants in the Whānau Pakari programme. Each point represents an individual participant. The horizontal bars indicate the lower quartile, median and upper quartile of the distribution. The values at the bottom of the plot show the proportion of participants who experienced a reduction in BMI SDS

**Fig. 2:**
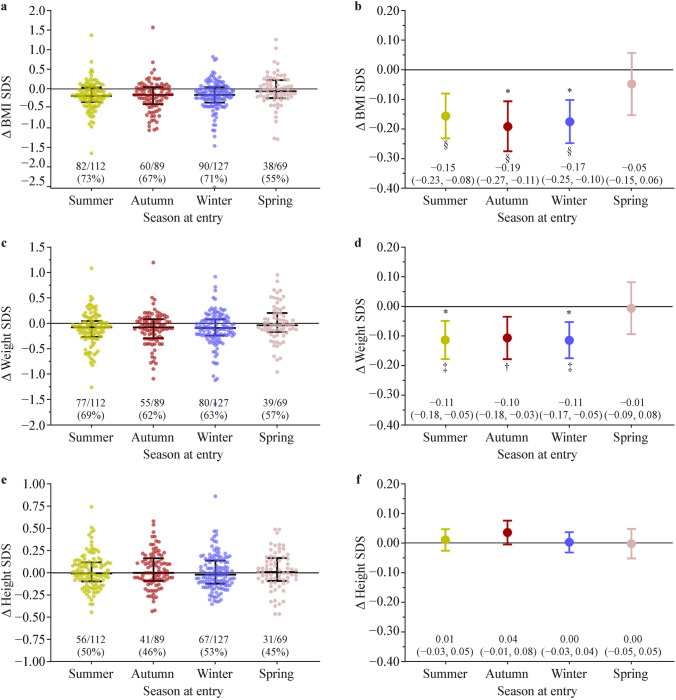
Anthropometric changes (Δ) in participants in the Whānau Pakari programme at the 6-month follow-up according to season at entry. Panels **a**, **c** and **e** are sinaplots showing changes in BMI standard deviation scores (SDS), weight SDS and height SDS respectively, for all study participants. The horizontal bars represent the lower quartiles, medians and upper quartiles, while the values provided are proportions of participants with a reduction in a given outcome. Panels **b**, **d** and **f** display respective adjusted means and 95% confidence intervals for each season derived from generalised linear mixed models adjusted for season at entry, cohort (service or trial), sex, ethnicity, age and baseline outcome value, as well as family ID as a random factor. *P < 0.05 for pairwise comparisons to spring entrants. ^†^*P* < 0.01, ^‡^*P* < 0.001, and ^§^*P* < 0.0001 for within-season differences compared to baseline

Two out of three participants (270/397; 68%) had a lower BMI SDS at 6 months (Fig. 1), with an overall mean reduction in BMI SDS of − 0.16 (95% CI − 0.20 to − 0.12; *P* < 0.0001). However, the proportion of participants with a BMI SDS reduction varied by season, from 55% in spring entrants to 73% in their summer counterparts (Fig. 2). Summer and winter entrants were 36% [aRR = 1.36 (95% CI 1.09–1.71); *P* = 0.009] and 32% [aRR = 1.32 (1.04–1.67); *P* = 0.021], respectively, more likely to display a BMI SDS reduction at 6 months compared to participants enrolled in spring.

Unadjusted data showed BMI SDS reductions in participants enrolled in summer (− 0.17 SDS; *P* < 0.0001), autumn (− 0.19 SDS; *P* < 0.0001) and winter (− 0.18 SDS; *P* < 0.0001), but not spring (*P* = 0.30) (Table 2). Compared to the spring enrolment group, BMI SDS reductions were greater for autumn [− 0.14 SDS (95% CI − 0.27 to − 0.01); *P* = 0.039] and winter [− 0.13 SDS (− 0.25 to − 0.01); *P* = 0.044] but not summer entrants [− 0.10 SDS (− 0.23 to 0.02); *P* = 0.11]. Greater BMI SDS at baseline correlated with a greater BMI SDS reduction [*ρ* = − 0.15 (95% CI − 0.24 to − 0.05); *P* = 0.004]. The opposite pattern was seen for age at entry [*ρ* = 0.16 (95% CI 0.06–0.26); *P* = 0.001].

**Table 2:** Changes (Δ) in anthropometric parameters at 6 months among study participants overall and according to the season at entry into the Whānau Pakari programme

Variables	Overall	Summer	Autumn	Winter	Spring
*n*	397	112	89	127	69
Δ height SDS	0.02 (0.00 to 0.04)*	0.02 (− 0.01 to 0.06)	0.03 (− 0.01 to 0.08)	0.01 (− 0.03 to 0.04)	0.02 (− 0.03 to 0.07)
Δ weight SDS^a^	− 0.09 (− 0.12 to − 0.06)^§^	− 0.11 (− 0.17 to − 0.05)^‡^^||^	− 0.11 (− 0.18 to − 0.04)^†||^	− 0.11 (− 0.17 to − 0.05)^‡||^	0.01 (− 0.08 to 0.09)
Δ BMI SDS	− 0.16 (− 0.20 to − 0.12)^§^	− 0.17 (− 0.24 to − 0.10)^§^	− 0.19 (− 0.27 to − 0.10)^§ ||^	− 0.18 (− 0.25 to − 0.10)^§ ||^	− 0.06 (− 0.17 to 0.05)
Δ WHtR	− 0.008 (− 0.011 to − 0.005)^§^	− 0.008 (− 0.014 to − 0.002)^†^	− 0.013 (− 0.019 to − 0.006)^‡||^	− 0.008 (− 0.014 to − 0.003)^†||^	− 0.002 (− 0.010 to 0.006)

*n*, sample size for a given outcome; all other data are unadjusted means (Δ) and 95% confidence intervals (CI). Within- and between-season Δ were assessed with paired *t*-tests and one-way ANOVA, respectively

Seasons were defined per meteorological criteria [25]

*BMI* body mass index, *SDS* standard deviation score, *WHtR* waist-to-height ratio

^*^P < 0.05, ^†^*P* < 0.01, ^‡^*P* < 0.001, and ^§^*P* < 0.0001 for an overall or within-season change from baseline, ^||^*P* < 0.05 for pairwise comparisons to spring entrants

^a^Negative values for Δ weight SDS indicate lower weight gain relative to age and sex, not absolute weight loss

Within-season changes seen in BMI SDS remained largely unaltered after adjustment for other factors in the multivariable model. There were reductions in summer (aMD = − 0.15 SDS; *P* < 0.0001), autumn (− 0.19 SDS; *P* < 0.0001) and winter (− 0.17 SDS; *P* < 0.0001) but not spring entrants (− 0.05 SDS; *P* = 0.37) (Fig. 2b). Similarly, compared to spring entrants, there were greater BMI SDS reductions among autumn [aMD = − 0.14 SDS (95% CI − 0.27 to − 0.01 SDS); *P* = 0.034] and winter [− 0.13 SDS (− 0.25 to 0.00 SDS); *P* = 0.049] but not summer [− 0.11 SDS (− 0.23 to 0.02 SDS); *P* = 0.09] entrants (Fig. 2b).

With respect to other factors in the multivariable model, a higher baseline BMI SDS was associated with a greater subsequent reduction in BMI SDS at 6 months [β = − 0.096 (95% CI − 0.137 to − 0.056); *P* < 0.0001]. Conversely, sex (*P* = 0.86), age (*P* = 0.13) and programme cohort (*P* = 0.72) were not associated with Δ BMI SDS.

Considering the unbalanced distribution of spring entrants according to programme cohort (90% were from the service; Table 1) an additional analysis was carried out based on the final multivariable model for Δ BMI SDS. This model included cohort and a season*cohort interaction term. There was no significant interaction (*P* = 0.85) or evidence of differential seasonal variations in Δ BMI SDS according to programme cohort (Supplementary Fig. 3).

Finally, exploratory analysis using the UK Cole reference, typically adopted in NZ clinics, showed attenuated results, with an overall mean BMI SDS reduction of − 0.10 SDS (95% CI − 0.13 to − 0.07 SDS; *P* < 0.0001) (Supplementary Fig. 4a, b). There were no detectable between-season differences in Δ BMI SDS in univariable (Supplementary Fig. 4c) or multivariable (Supplementary Fig. 4d) models (both *P* > 0.28 overall; for all pairwise comparisons *P* > 0.07). However, there was evidence that BMI SDS decreased among summer, autumn and winter but not spring entrants; there were negligible differences in estimates from unadjusted and adjusted models (Supplementary Fig. 4c, d).

### Other anthropometric outcomes

There were decreases in weight SDS at 6 months that largely mirrored the BMI SDS patterns, indicating lower weight gain relative to age and sex, with an overall change of − 0.09 SDS (95% CI − 0.12 to − 0.06; *P* < 0.0001). This was observed among summer (− 0.11 SDS; *P* = 0.0005), autumn (− 0.11 SDS; *P* = 0.003) and winter (− 0.11 SDS; *P* = 0.0004) but not spring (*P* = 0.88) entrants (Table 2; Fig. 2c). The magnitude of the weight SDS reductions were unchanged in the multivariable model (Fig. 2d). In comparison to spring entrants, there were identical weight reductions in summer and winter entrants [both aMD = − 0.11 SDS (95% CI − 0.21 to 0.00); *P* = 0.048 and *P* = 0.049, respectively], with a similar trend in the autumn group [− 0.10 SDS (− 0.21 to 0.01); *P* = 0.078] (Fig. 2d).

There was also evidence of a lower waist-to-height ratio among summer, autumn and winter entrants in both univariable (Table 2) and multivariable models [aMD = − 0.006 (95% CI − 0.012 to 0.000); *P* = 0.044], [− 0.011 (− 0.018 to − 0.005); *P* = 0.0006], and [− 0.009 (− 0.015 to − 0.004); *P* = 0.001], respectively. Compared to spring entrants, reductions were greater for autumn [aMD = − 0.013 (95% CI − 0.023 to − 0.002); *P* = 0.014] and winter [− 0.011 (− 0.020 to − 0.001); *P* = 0.034] but not summer (*P* = 0.21) entrants. Conversely, despite a marginal change in height SDS overall [+ 0.02 SDS (95% CI 0.00–0.04); *P* = 0.039], there were no observed within- or between-season differences in unadjusted (Table 2) and adjusted models (Fig. 2e, f).

### Dietary factors and lifestyle

At baseline, most dietary and lifestyle factors were similar between seasons (Supplementary Table 1). The exception was sleep duration (*P* = 0.023), as spring (median difference = + 30 min; *P* = 0.031) and winter (+ 30 min; *P* = 0.007) entrants reported slightly longer sleep duration compared to autumn entrants, with a similar trend for winter vs summer entrants (+ 15 min; *P* = 0.053) (Supplementary Table 1).

At 6 months, median sweet drink consumption was 30 mL/day lower (*P* < 0.0001; Table 3); 57% of participants (220/389) reported reducing their intake, with 35% (*n* = 138) and 26% (*n* = 100) reporting reductions ≥ 100 and ≥ 200 mL/day, respectively. Participants also reported increased physical activity levels (median = + 13 min/day; *P* = 0.0006) and a marginal reduction in screen time (median =  − 9 min/day; *P* = 0.028) (Table 3). There was also limited evidence of other dietary improvement, as 41% of participants reported increasing their intake of fruit and vegetables by one or more servings per day (*P* = 0.012) (Table 3).

**Table 3:** Changes (Δ) in self-reported dietary and lifestyle factors at 6 months in study participants according to the season of entry into the Whānau Pakari programme

Lifestyle factor	Overall	Summer	Autumn	Winter	Spring	*P*
Perceived physical activity						
*n*	359	100	81	117	61	
Δ (min/day)	13 (− 29, 52)^‡^	3 (− 45, 34)^¶^	15 (− 36, 50)^||^	19 (− 9, 66)^§^	8 (− 28, 52)^||^	**0.008**
Time spent on screens						
*n*	363	102	82	118	61	
Δ (min/day)	− 9 (− 71, 51)*	6 (− 51, 64)^||^	− 2 (− 69, 49)	− 19 (− 111, 35)^†^	− 17 (− 51, 56)	0.15
Sweet drink intake^a^						
*n*	389	111	87	122	69	
Δ (mL/day)	− 30 (− 201, 24)^§^	− 36 (− 250, 0)^§^	− 24 (− 179, 12)^†^	− 12 (− 179, 43)^†^	− 36 (− 250, 35)^†^	0.75
Fruit/vegetable consumption						
*n*	346	101	84	117	66	
Δ (servings/day)	0 (− 1.0, 1.0)*	0 (0.0, 1.5)*	0 (− 1.0, 2.0)	0 (− 1.0, 1.0)	0 (− 1.0, 1.0)	0.38
Sleep duration						
*n*	379	106	82	124	67	
Δ (min/day)	0 (− 30, 30)	0 (− 30, 30)	0 (− 30, 30)	0 (− 60, 30)*	0 (− 60, 30)	0.34

*n*, the sample size for a given outcome. Data are reported as the unadjusted median (lower quartile, upper quartile). Within-season Δ were assessed with non-parametric Wilcoxon signed-rank tests, where *P < 0.05, ^†^*P* < 0.01, ^‡^*P* < 0.001, and ^§^*P* < 0.0001. Between-season Δ differences were analysed using ranked generalised linear mixed models adjusted for season at entry, cohort (service/trial), sex (male/female), ethnicity (Māori/Non-Māori), age and the ranked baseline outcome value, as well as family ID as a random factor. The *P* value derived from each multivariable model for the overall seasonal effect is provided and shown in bold if statistically significant at *P* < 0.05; pairwise effect magnitudes were quantified using Wilcoxon-derived Hodges–Lehmann location shift estimates with 95% confidence intervals, with ^||^*P* < 0.05 and ^¶^*P* < 0.001 for differences in comparison to Winter entrants. Seasons were defined per meteorological criteria [25]

^a^Sweet drinks included powdered drinks, cordial, fruit juice, energy drinks, and carbonated sweet drinks

When seasonal changes were assessed, sweet drink intake was reduced across all groups independently of season at entry (all *P* < 0.006) (Table 3). Winter entrants reported a median increase in physical activity of 19 min/day (*P* < 0.0001; Table 3), which was greater than that reported by summer [median difference = + 33 min/day (95% CI 15–50); *P* = 0.0008], autumn [+ 19 min/day (0–38); *P* = 0.045] and spring [+ 16 min/day (− 2, 37); *P* = 0.047] entrants. In addition, there was a greater increase in screen time in summer compared to winter entrants [median difference = 31 min/day (95% CI 4–61); *P* = 0.030].

### Random forest

**Fig. 3:**
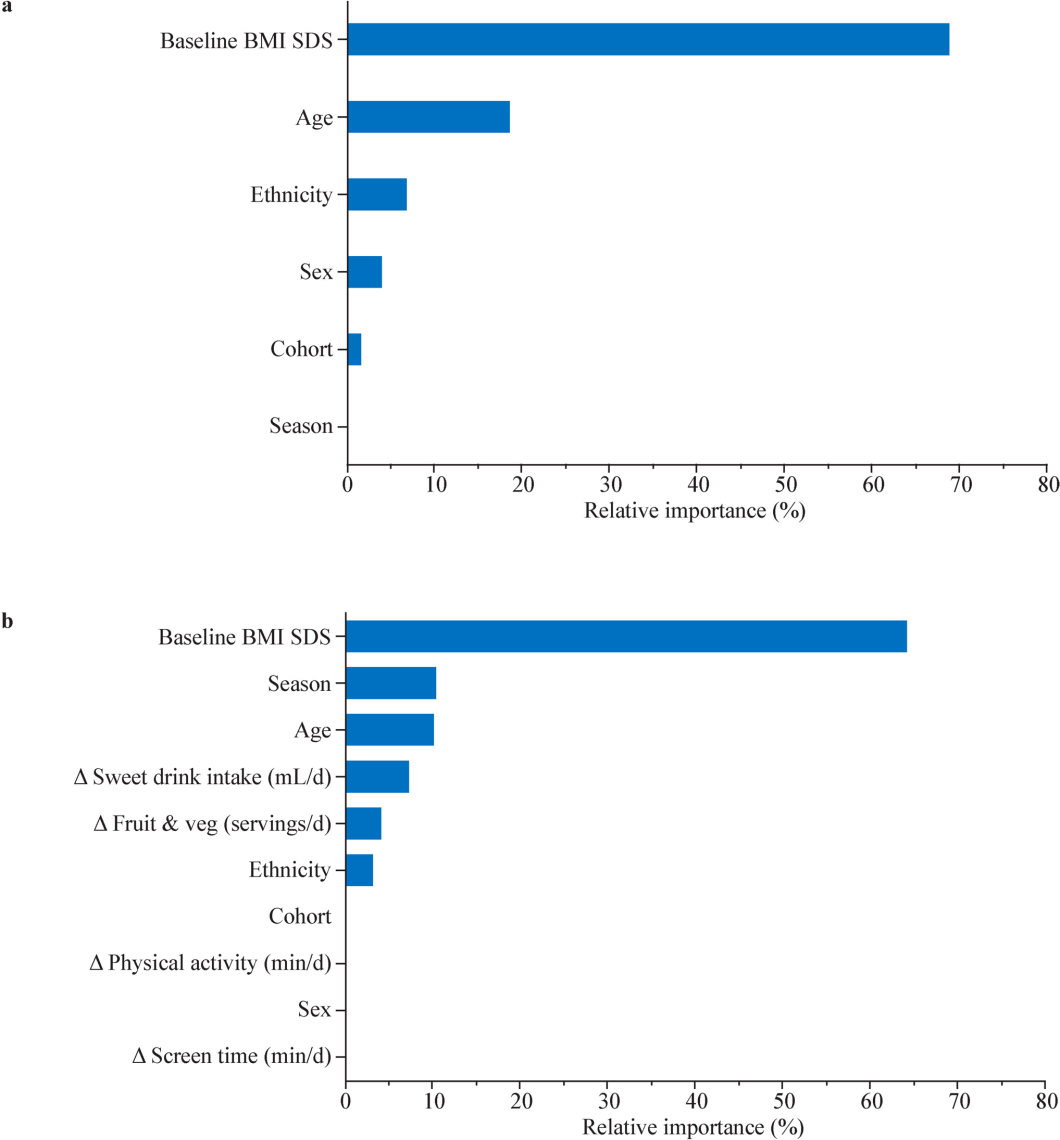
Random forest analysis of the relative importance of predictors for 6-month change in BMI standard deviation scores (SDS). Panel **a** displays the base model, which included demographic and clinical variables (baseline BMI SDS, age, ethnicity, sex, cohort and season). Panel **b** displays the expanded model, including all variables from the base model plus 6-month changes (Δ) in self-reported diet and lifestyle factors. Relative importance indicates which variables were most useful for predicting BMI SDS change; larger values reflect larger drops in accuracy when the variable was randomly shuffled (scaled to sum to 100%)

The random forest base model, which included only demographic and clinical variables, showed that higher baseline BMI SDS (68.9% relative importance) and younger age (18.7%) were the strongest predictors of greater reductions in BMI SDS (Fig. 3a). In this model, season of entry had no relative importance (0.0%) (Fig. 3a).

However, when changes in diet and lifestyle were included, the relative predictor importance shifted markedly (Fig. 3b). While baseline BMI SDS (64.2%) and age (10.3%) remained as the primary predictors, season of entry emerged as the second most important variable (10.5%), ahead of changes in sweet drink intake (7.4%) and fruit and vegetable consumption (4.3%) (Fig. 3b). Among demographic factors, ethnicity had low relative importance (3.3%), while sex and cohort had no relative importance (both 0.0%) (Fig. 3b). Model summaries indicated that younger age and, especially, higher baseline BMI SDS were associated with greater predicted reductions in BMI SDS at 6 months (Supplementary Fig. 5). In the expanded model, the mean 6-month Δ BMI SDS predicted for spring entrants was − 0.10 (95% CI − 0.16 to − 0.04); this remained attenuated compared with the predicted Δ BMI SDS for summer (− 0.17; − 0.20 to − 0.13), autumn (− 0.17; − 0.22 to − 0.13) and winter (− 0.17; − 0.21 to − 0.13) entrants.

## Discussion

We observed seasonal differences in the effectiveness of a multidisciplinary childhood obesity intervention programme in NZ. Specifically, spring entrants showed attenuated BMI SDS reductions. We speculate that this attenuated effectiveness for the spring cohort was linked to the 5–7-week summer school holidays, which fall entirely within their intervention period, as per the “Structured Days Hypothesis” [11]. Brusseau et al. showed that children in schools with short 3-week breaks at frequent intervals throughout the year did not experience weight gain in the summer compared with children from schools with longer 12-week summer breaks [33]. The rise in less healthy behaviour is thought to be a major reason for weight gain during unstructured holiday breaks due to fewer restrictions and planned times for meals, physical activity, screen time and sleep [11]. In the case of Whānau Pakari, the associated minimal or no contact during this time likely led to a marked break in engagement, negatively affecting intervention outcomes for this group. A U.S. study reported similar findings that summer breaks (without programme supervision) were associated with increased adiposity and lower fitness gains [8]. Interestingly, participants commencing Whānau Pakari in December (summer entrants with less initial contact but more sustained programme support after the summer holidays) showed BMI SDS reductions at six months, unlike spring recruits.

Overall, our multidisciplinary programme was associated with improvements in several self-reported dietary and lifestyle behaviours irrespective of the entry season. These included reduced intake of sweet drinks, increased physical activity levels, decreased screen time and higher fruit or vegetable consumption. However, there was no evidence of consistent seasonal differences in self-reported dietary and lifestyle behaviours at 6 months.

Importantly, our random forest analyses provided a more refined view of these relationships. In a model without lifestyle factors, season had no predictive power and its effect was likely masked by other sources of variation. However, once self-reported changes in diet and lifestyle were accounted for, season of entry emerged as a key predictor of greater importance than any single lifestyle factor. This suggests that season is a strong proxy for the unique circumstances of spring entrants (likely related to the summer holidays), whose attenuated outcomes are not fully explained by the measured lifestyle variables alone. Persistence of the importance of season after including these factors suggests that it captures a broader range of unmeasured influences associated with the impending summer holiday period; this is consistent with the “Structured Days Hypothesis” [11].

The Whānau Pakari programme involved 6 months of weekly group sessions after the trial. This included offering home-based assessments, follow-ups and weekly one-hour group sessions delivered during school terms, consistent with the U.S. Preventive Services Task Force recommended minimum contact time of ≥ 26 hours for effectiveness [19]. Our study suggests that evaluating multidisciplinary interventions at a single 6-month time point may be susceptible to seasonal bias and may not reliably assess programme efficacy. Overlooked seasonal factors could compromise the accuracy of trials reporting only short-term (≤ 6 months) outcomes for healthy lifestyle interventions, as behavioural shifts during long unstructured holidays or due to climatic factors (especially at higher latitudes) could add bias.

The magnitude of the observed seasonal differences in Δ BMI SDS (0.11–0.13 SDS) warrants consideration, given their potential clinical relevance in the context of healthy lifestyle interventions. From a service delivery perspective, multidisciplinary healthy lifestyle programmes such as Whānau Pakari need to consider whether holiday sessions should be run to minimise the effect of the long summer holiday on spring entrants. If spring entrants are less likely to achieve a BMI SDS reduction after six months, they may feel discouraged and less motivated to make and maintain long-term healthy lifestyle changes, as previously reported in a qualitative study [34]. Of note, study participants with higher baseline BMI SDS showed a greater reduction in BMI SDS at six months. This association was seen in a simple correlation, a multivariable model and random forest analysis. Similarly, a recent systematic review and network meta-analysis also reported that children with higher baseline BMI experienced a larger effect size (i.e., greater BMI reduction) in response to lifestyle interventions [35].

The main limitation of our study was the lack of 12-month follow-up data for all participants, which would allow the examination of longer-term seasonal variations in BMI SDS and the sustainability of the changes seen at 6 months. Another limitation was our inability to fully control for socioeconomic deprivation in our statistical analyses, since this information was only available for a proportion of the participants; this may have introduced residual confounding. In addition, lifestyle factors were self- or proxy-reported; these assessments of dietary intake and physical activity levels in children and adolescents are susceptible to bias [36, 37]. An important consideration was whether differences between the trial and the subsequent clinical service drove the observed seasonal influences. Formal interaction testing in our statistical models showed that seasonal influences were largely homogeneous in the two cohorts. Furthermore, our random forest analysis consistently identified programme cohort as a predictor of very limited importance compared to several other factors, including season itself. Therefore, these analyses provide evidence that the observed seasonal variations in outcomes were likely genuine. Our data were from a real-world, multidisciplinary intervention programme, with a strong representation of children and adolescents most affected by obesity in NZ; the integration of trial and service data strengthens the potential generalisability of our findings.

While the observed mean reduction in BMI SDS (− 0.16 SDS overall) across our study population may not meet higher thresholds proposed for reversing obesity (e.g., − 0.6 SDS [38]), it is still clinically important in pediatric weight management, where slowing or halting the expected upward trajectory of BMI SDS is a primary therapeutic goal. Importantly, we showed that these BMI SDS reductions were driven by changes in weight trajectory relative to age and sex (Δ weight SDS) rather than by accelerated linear growth (Δ height SDS). While a minority of participants achieved modest absolute weight loss, the primary mechanism underlying the BMI SDS reductions was an attenuation of relative weight gain. This outcome aligns with key therapeutic goals for pediatric obesity management, which seek to improve body proportionality and reduce relative adiposity by moderating excessive weight gain in growing children [39]. The concurrent stability in height SDS provides reassurance that our healthy lifestyle intervention was beneficial and did not compromise linear growth, addressing an important concern that some interventions targeting absolute weight loss can impair growth [39]. Furthermore, the success of Whānau Pakari's healthy lifestyle intervention is also defined by several positive behavioural changes, including reduced sweet drink intake, increased physical activity and decreased screen time. The clinical importance of our findings is twofold, encompassing both the modest but favourable impact on BMI SDS and positive changes in health-promoting behaviours.

In conclusion, our findings show that the season of programme entry is likely an important factor associated with the effectiveness of childhood obesity interventions in NZ. The attenuated success for spring entrants highlights the challenges posed by the unstructured summer holiday period that occurs during the intervention period. Our machine learning analysis suggests this seasonal influence is complex and not fully explained by individual lifestyle changes alone. This reinforces the need to consider the broader context of a child's environment when designing and evaluating interventions. If seasonal dynamics are not considered, short-term outcome assessments may mischaracterise the true intervention effect; robust evaluation therefore requires longer follow-up, ideally at least 12 months. Future work should focus on developing strategies, such as enhanced holiday support, to mitigate these seasonal effects and ensure all participants have an equal opportunity for success.

## Supplementary Information

Below is the link to the electronic supplementary material.

Supplementary file1 (PDF 487 KB)

## Data Availability

Due to the confidential and sensitive nature of the clinical audit data, public access to this dataset is restricted in accordance with the protection of participant health information. Explicit consent was not obtained during the audit to share this dataset. Similarly, the dataset from the Whānau Pakari randomised clinical trial is not publicly available, owing to the stringent conditions set forth by the ethics approval. Nevertheless, access to both datasets may be granted upon reasonable request, contingent upon receiving full ethics approval for the proposed study protocol and statistical analysis plan.

## Declarations

### Use of artificial intelligence

JGB Derraik used Claude 4.1 Opus (Anthropic, San Francisco, CA, USA) to assist with “debugging” and refinement of SAS code, and M Shahbaz used ChatGPT 5 “Thinking” (OpenAI, San Francisco, CA, USA) to assist with refinement of *R* code. JGB Derraik also used ChatGPT 5 “Thinking” and Gemini 2.5 Pro (Google LLC, Mountain View, CA, USA) for critical editorial review to improve text clarity and accuracy, and for final proofreading. Following use of these tools, the authors critically reviewed and edited the content as needed, and take full responsibility for the manuscript's content.

## References

[1] UNICEF. The State of the World’s Children 2019. Children, Food and Nutrition: Growing Well in a Changing World. New York: UNICEF; 2019.

[2] Ministry of Health. Annual Data Explorer 2023/24: New Zealand Health Survey [Data file]. Wellington (NZ): Ministry of Health; 2024. Available from: https://www.health.govt.nz/publications/annual-update-of-key-results-202324-new-zealand-health-survey. Accessed 7 Dec 2024.

[3] Chu DT, Minh Nguyet NT, Dinh TC, Thai Lien NV, Nguyen KH, Nhu Ngoc VT, et al. An update on physical health and economic consequences of overweight and obesity. Diabetes Metab Syndr. 2018;12:1095–100. 29799416 10.1016/j.dsx.2018.05.004

[4] Bhutani S, Wells N, Finlayson G, Schoeller DA. Change in eating pattern as a contributor to energy intake and weight gain during the winter holiday period in obese adults. Int J Obes. 2020;44:1586–95. 10.1038/s41366-020-0562-2 PMC7332403 32203107

[5] Shahar DR, Froom P, Harari G, Yerushalmi N, Lubin F, Kristal-Boneh E. Changes in dietary intake account for seasonal changes in cardiovascular disease risk factors. Eur J Clin Nutr. 1999;53:395–400. 10369496 10.1038/sj.ejcn.1600761

[6] Tanskey LA, Goldberg JP, Chui K, Must A, Sacheck JM. Accelerated summer weight gain in a low-income, ethnically diverse sample of elementary school children in Massachusetts. Child Obes. 2019;15:244–53. 30888836 10.1089/chi.2017.0228 PMC6909732

[7] Brusseau TA, Burns RD. Children’s weight gain and cardiovascular fitness loss over the summer. Int J Environ Res Public Health. 2018;15:2770. 30544487 10.3390/ijerph15122770 PMC6313671

[8] Carrel AL, Clark RR, Peterson S, Eickhoff J, Allen DB. School-based fitness changes are lost during the summer vacation. Arch Pediatr Adolesc Med. 2007;161:561–4. 17548760 10.1001/archpedi.161.6.561

[9] Franckle R, Adler R, Davison K. Accelerated weight gain among children during summer versus school year and related racial/ethnic disparities: a systematic review. Prev Chronic Dis. 2014;11:E101. 24921899 10.5888/pcd11.130355 PMC4060873

[10] Gillis L, McDowell M, Bar-Or O. Relationship between summer vacation weight gain and lack of success in a pediatric weight control program. Eat Behav. 2005;6:137–43. 15598600 10.1016/j.eatbeh.2004.08.002

[11] Brazendale K, Beets MW, Weaver RG, Pate RR, Turner-McGrievy GM, Kaczynski AT, et al. Understanding differences between summer vs. school obesogenic behaviors of children: the Structured Days Hypothesis. Int J Behav Nutr Phys Act. 2017;14:100. 28747186 10.1186/s12966-017-0555-2 PMC5530518

[12] Ridgers ND, Salmon J, Timperio A. Too hot to move? Objectively assessed seasonal changes in Australian children’s physical activity. Int J Behav Nutr Phys Act. 2015;12:77. 26088561 10.1186/s12966-015-0245-x PMC4479354

[13] Nixon GM, Thompson JM, Han DY, Becroft DM, Clark PM, Robinson E, et al. Short sleep duration in middle childhood: risk factors and consequences. Sleep. 2008;31:71–8. 18220080 10.1093/sleep/31.1.71 PMC2225560

[14] Taylor RW, Murdoch L, Carter P, Gerrard DF, Williams SM, Taylor BJ. Longitudinal study of physical activity and inactivity in preschoolers: the FLAME study. Med Sci Sports Exerc. 2009;41:96–102. 19092702 10.1249/MSS.0b013e3181849d81

[15] Ergler CR, Kearns RA, Witten K. Seasonal and locational variations in children’s play: implications for wellbeing. Soc Sci Med. 2013;91:178–85. 23312793 10.1016/j.socscimed.2012.11.034

[16] Anderson YC, Wynter LE, Moller KR, Cave TL, Dolan GM, Grant CC, et al. The effect of a multi-disciplinary obesity intervention compared to usual practice in those ready to make lifestyle changes: design and rationale of Whanau Pakari. BMC Obes. 2015;2:41. 26464806 10.1186/s40608-015-0068-y PMC4599755

[17] Anderson YC, Wynter LE, Grant CC, Cave TL, Derraik JGB, Cutfield WS, et al. A novel home-based intervention for child and adolescent obesity: the results of the Whānau Pakari randomized controlled trial. Obesity. 2017;25:1965–73. 29049868 10.1002/oby.21967

[18] Anderson YC, Wynter LE, O’Sullivan NA, Wild CEK, Grant CC, Cave TL, et al. Two-year outcomes of Whānau Pakari, a multi-disciplinary assessment and intervention for children and adolescents with weight issues: a randomized clinical trial. Pediatr Obes. 2021;16:e12693. 32959996 10.1111/ijpo.12693

[19] Whitlock EP, O'Connor EA, Williams SB, Beil TL, Lutz KW. Effectiveness of primary care interventions for weight management in children and adolescents: an updated, targeted systematic review for the USPSTF. Rockville: Agency for Healthcare Research and Quality (US); 2010. 20722175

[20] Cole TJ, Freeman JV, Preece MA. Body mass index reference curves for the UK, 1990. Arch Dis Child. 1995;73:25–9. 7639544 10.1136/adc.73.1.25 PMC1511150

[21] Anderson YC, Dolan GMS, Wynter LE, Treves KF, Wouldes TA, Grant CC, et al. Caregiver’s readiness for change as a predictor of outcome and attendance in an intervention programme for children and adolescents with obesity: a secondary data analysis. BMJ Open. 2019;9:e023195. 30918030 10.1136/bmjopen-2018-023195 PMC6475337

[22] Ministry of Health. HISO 10001:2017—Ethnicity Data Protocols, Version 1.1. Wellington: Health Information Standards Organisation, Ministry of Health; 2017.

[23] Magarey A, Golley R, Spurrier N, Goodwin E, Ong F. Reliability and validity of the Children’s Dietary Questionnaire; a new tool to measure children’s dietary patterns. Int J Pediatr Obes. 2009;4:257–65. 19922040 10.3109/17477160902846161

[24] Corder K, van Sluijs EM, Wright A, Whincup P, Wareham NJ, Ekelund U. Is it possible to assess free-living physical activity and energy expenditure in young people by self-report? Am J Clin Nutr. 2009;89:862–70. 19144732 10.3945/ajcn.2008.26739

[25] Trenberth KE. What are the seasons? Bull Am Meteorol Soc. 1983;64:1276–82.

[26] Zou G. A modified poisson regression approach to prospective studies with binary data. Am J Epidemiol. 2004;159:702–6. 15033648 10.1093/aje/kwh090

[27] Barnett MJ, Doroudgar S, Khosraviani V, Ip EJ. Multiple comparisons: to compare or not to compare, that is the question. Res Social Adm Pharm. 2022;18:2331–4. 34274218 10.1016/j.sapharm.2021.07.006

[28] Rothman KJ. No adjustments are needed for multiple comparisons. Epidemiology. 1990;1:43–6. 2081237

[29] Domb BG, Sabetian PW. The blight of the type II error: when no difference does not mean no difference. Arthroscopy. 2021;37:1353–6. 33581304 10.1016/j.arthro.2021.01.057

[30] Breiman L. Random forests. Mach Learn. 2001;45:5–32.

[31] Cutler DR, Edwards TC Jr., Beard KH, Cutler A, Hess KT, Gibson J, et al. Random forests for classification in ecology. Ecology. 2007;88:2783–92. 18051647 10.1890/07-0539.1

[32] *R* Core Team. *R*: a language and environment for statistical computing. Vienna: *R* Foundation for Statistical Computing; 2024. https://www.R-project.org/.

[33] Brusseau TA, Burns RD, Fu Y, Weaver RG. Impact of year-round and traditional school schedules on summer weight gain and fitness loss. Child Obes. 2019;15:541–7. 31364859 10.1089/chi.2019.0070 PMC9208378

[34] Wild CE, Rawiri NT, Willing EJ, Hofman PL, Anderson YC. Challenges of making healthy lifestyle changes for families in Aotearoa/New Zealand. Public Health Nutr. 2021;24:1906–15. 33155537 10.1017/S1368980020003699 PMC8094428

[35] Su X, Hassan MA, Kim H, Gao Z. Comparative effectiveness of lifestyle interventions on children’s body composition management: a systematic review and network meta-analysis. J Sport Health Sci. 2025;14:101008. 39510316 10.1016/j.jshs.2024.101008 PMC11863321

[36] Adamo KB, Prince SA, Tricco AC, Connor-Gorber S, Tremblay M. A comparison of indirect versus direct measures for assessing physical activity in the pediatric population: a systematic review. Int J Pediatr Obes. 2009;4:2–27. 18720173 10.1080/17477160802315010

[37] Burrows T, Goldman S, Rollo M. A systematic review of the validity of dietary assessment methods in children when compared with the method of doubly labelled water. Eur J Clin Nutr. 2020;74:669–81. 31391548 10.1038/s41430-019-0480-3

[38] Birch L, Perry R, Hunt LP, Matson R, Chong A, Beynon R, et al. What change in body mass index is associated with improvement in percentage body fat in childhood obesity? A meta-regression. BMJ Open. 2019;9:e028231. 31473614 10.1136/bmjopen-2018-028231 PMC6720247

[39] Reinehr T. Lifestyle intervention in childhood obesity: changes and challenges. Nat Rev Endocrinol. 2013;9:607–14. 23897171 10.1038/nrendo.2013.149

