# Supplementary material for: Seasonal variation in BMI outcomes at 6 months: secondary analyses of a multidisciplinary healthy lifestyle programme for children and adolescents with obesity
Source: World J Pediatr. 2026 Feb 6;22(2):234–46. doi: 10.1007/s12519-025-01016-z (PMC12923471; doi:10.1007/s12519-025-01016-z)
Supplement: Supplementary file 1 — Supplementary file1 (PDF 487 KB) [file 12519_2025_1016_MOESM1_ESM.pdf]

SUPPLEMENTARY FILE

Seasonal variation in BMI outcomes at 6 months: secondary analyses of a multidisciplinary healthy lifestyle programme for children and adolescents with obesity

José G. B. Derraik[1,2†], Kima T. Costelloe[1,†], Cervantée E. K. Wild[1], Lisa E. Wynter[3], Mohammad Shahbaz[4], Paul L. Hofman[4], Yvonne C. Anderson[1,5,6,7,*]

[1] Department of Paediatrics, Child and Youth Health, Faculty of Medical and Health Sciences, University of Auckland, Auckland, New Zealand.

[2] Environmental–Occupational Health Sciences and Non-Communicable Diseases Research Centre, Research Institute for Health Sciences, Chiang Mai University, Chiang Mai, Thailand.

[3] Department of Paediatrics, Health New Zealand | Te Whatu Ora Taranaki, New Plymouth, New Zealand.

[4] Liggins Institute, University of Auckland, Auckland, New Zealand.

[5] Curtin Medical School, Faculty of Health Sciences, Curtin University, Bentley, Perth, WA, Australia.

[6] The Kids Research Institute Australia, Perth, WA, Australia.

[7] Child and Adolescent Community Health, Child and Adolescent Health Service, Perth, WA, Australia.

[†] These authors share first authorship.

*Author for correspondence: Professor Yvonne Anderson, Faculty of Health Sciences, Curtin University, Kent Street, Bentley, Perth, WA 6102, Australia. yvonne.anderson@curtin.edu.au

Supplementary Methods

Standardisation of Anthropometric Data

Participants were initially screened in clinic using the UK 1990 (UK90 or 'UK Cole') growth reference [1]. For this study, all anthropometric measurements were converted to age- and sex-specific standard deviation scores (SDS or *z*-scores) using the age-appropriate WHO growth system: the WHO 2006 Child Growth Standards for children <5 years [2] and the WHO 2007 Growth Reference for those 5–19 years [3]. The WHO 0–5 y Standards are prescriptive, representing how children should grow under optimal conditions [2]. These Standards were based on data from healthy breastfed children in six countries [2], and help identify deviations from optimal growth patterns in early childhood [4]. The WHO 5–19 y Reference is descriptive, constructed to join smoothly to the Standards at 5 years and to align with adult BMI cut-offs at 19 years [3]. Together, they form a harmonised WHO growth system spanning birth to 19 years, enabling *z*-score-based assessments across childhood and adolescence, and designed to transition smoothly at age 5 [2,3]. In contrast, the UK90 growth reference is descriptive rather than prescriptive, based on UK population-based cross-sectional growth surveys in 1978–1990 [1], which reflects how children grew prior to the obesity epidemic.

Our study population consisted of young people with obesity, mostly severe obesity. In this upper tail of the BMI distribution, different growth references can produce divergent *z*-scores for the same measurements because each reference mathematically models age-related changes differently [5]; specifically, how the distribution shape (L), median values (M), and variability (S) change as children grow. Consequently, very high BMI values can become compressed into a narrow *z*-score range across commonly used charts, which limits longitudinal discrimination, particularly for severe paediatric obesity [6,7].

Empirically in our data, the WHO growth system yielded larger and more widely spread BMI SDS values at the upper end of the spectrum than the UK90, allowing clearer longitudinal tracking and ranking among children with severe obesity (Supplementary Figure 1). Therefore, the WHO growth system was adopted for all inferential analyses, and we present limited UK90 data for context only. While some have cautioned that prescriptive standards should be used carefully for population surveillance [8], their use here provides a common benchmark for interpreting within-study changes, and facilitates international comparison of potential intervention effects against a consistent physiological growth target [9].

Supplementary Methods References

1. Cole TJ, Freeman JV, Preece MA. Body mass index reference curves for the UK, 1990. Arch Dis Child 1995; 73: 25–9.
2. WHO Multicentre Growth Reference Study Group, de Onis M. WHO Child Growth Standards based on length/height, weight and age. Acta Paediatr 2006; 95: 76–85.
3. de Onis M, Onyango AW, Borghi E, Siyam A, Nishida C, Siekmann J. Development of a WHO growth reference for school-aged children and adolescents. Bull World Health Organ 2007; 85: 660–67.
4. Grummer-Strawn LM, Reinold C, Krebs NF. Use of World Health Organization and CDC growth charts for children aged 0-59 months in the United States. MMWR Recomm Rep 2010; 59: 1–15.
5. de Onis M, Garza C, Onyango AW, Borghi E. Comparison of the WHO child growth standards and the CDC 2000 growth charts. J Nutr 2007; 137: 144–8.
6. Freedman DS, Butte NF, Taveras EM, Lundeen EA, Blanck HM, Goodman AB, Ogden CL. BMI *z*-scores are a poor indicator of adiposity among 2- to 19-year-olds with very high BMIs, NHANES 1999-2000 to 2013-2014. Obesity 2017; 25: 739–46.
7. Gulati AK, Kaplan DW, Daniels SR. Clinical tracking of severely obese children: a new growth chart. Pediatrics 2012; 130: 1136–40.
8. Cameron N, Hawley NL. Should the UK use WHO growth charts? Paediatr Child Health 2010; 20: 151–6.
9. Moon JS. Reappraisal of regional growth charts in the era of WHO growth standards. Pediatr Gastroenterol Hepatol Nutr 2013; 16: 137–42.

Supplementary Figure 1

Comparison of baseline body mass index standard deviation scores (BMI SDS) calculated using the UK 1990 (UK90) growth reference and the WHO growth system (WHO 0–5 y Child Growth Standards; WHO 2007 Growth Reference 5–19 y) among Whānau Pakari participants.

(a) Distribution of BMI SDS at baseline – For each participant, BMI SDS was standardised to age- and sex-specific values using: (i) the UK 1990 (UK90) reference [1] or (ii) the age-appropriate WHO growth system (either the WHO Child Growth Standards for children <5 years [2] or the WHO 2007 Growth Reference for those ≥5 years [3]). The horizontal bars indicate the lower quartile, median, and upper quartile of the distribution. The UK90 distribution appears more compressed at the upper tail, where the WHO and UK90 curves diverge.

(b) Paired comparison of BMI SDS – Each point is one child: *x*-axis = UK90 and *y*-axis = WHO (age-appropriate growth system), standardised to age- and sex-specific values, with children aged <5 years plotted as blue open triangles [2] and those ≥5 years in black circles [3]; the grey line is the identity line (where *x* = *y*). Points above the line indicate higher SDS under WHO than UK90, and the upward deviation at high SDS illustrates the ceiling effect of UK90, where large increases in BMI translate into smaller SDS than under WHO.

Note that the WHO provides a ***standard*** for ages ***0–5*** years (prescriptive) and a ***reference*** for ***5–19 years*** (descriptive). Therefore, the term ***WHO growth system*** is used to indicate that the age-appropriate WHO component was applied to each child.

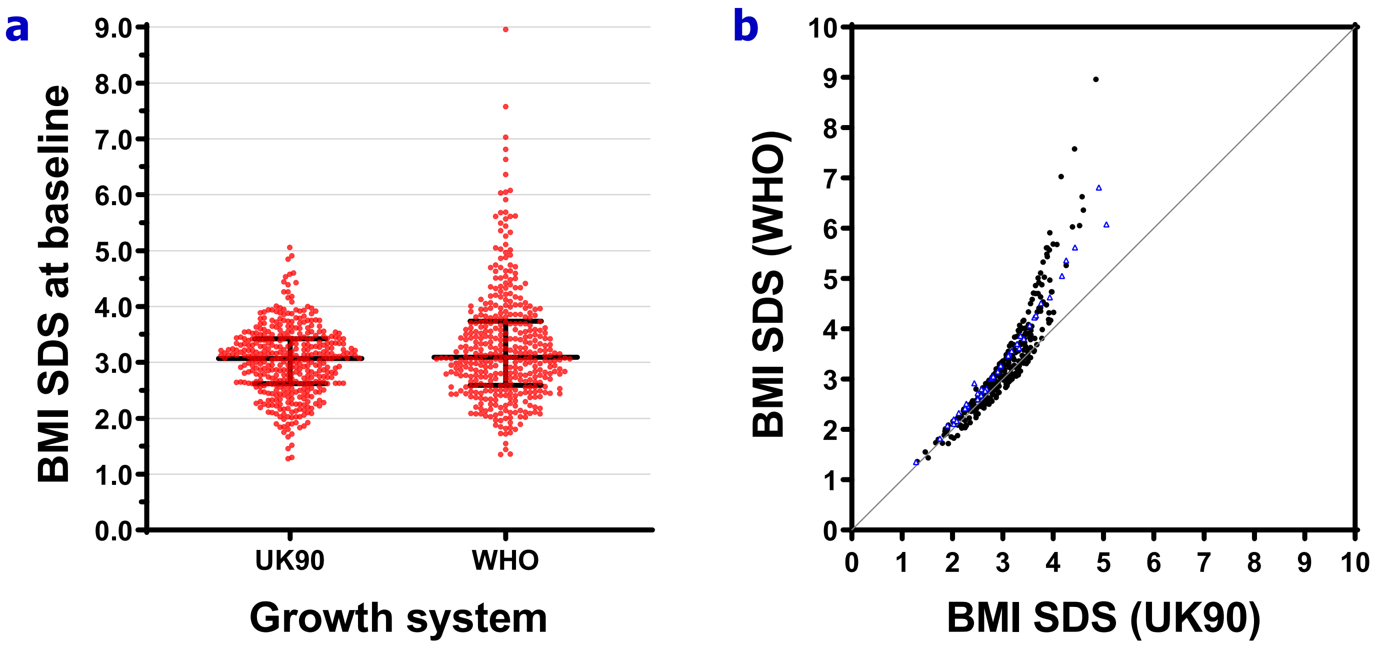

Supplementary Figure 1 References

1. Cole TJ, Freeman JV, Preece MA. Body mass index reference curves for the UK, 1990. Arch Dis Child 1995; 73: 25–9.
2. WHO Multicentre Growth Reference Study Group, de Onis M. WHO Child Growth Standards based on length/height, weight and age. Acta Paediatr 2006; 95: 76–85.
3. de Onis M, Onyango AW, Borghi E, Siyam A, Nishida C, Siekmann J. Development of a WHO growth reference for school-aged children and adolescents. Bull World Health Organ 2007; 85: 660–7.

Supplementary Figure 2

Sinaplots showing the distribution of the participants' ages (Panel *a*) and body mass index standard deviation scores (BMI SDS; Panel *b*) at baseline according to the season of entry into the Whānau Pakari programme.

The horizontal bars indicate the lower quartile, median, and upper quartile of the distribution. Seasons were defined per meteorological criteria (Trenberth KE, Bull Am Meteorol Soc 1983;64:1276–82).

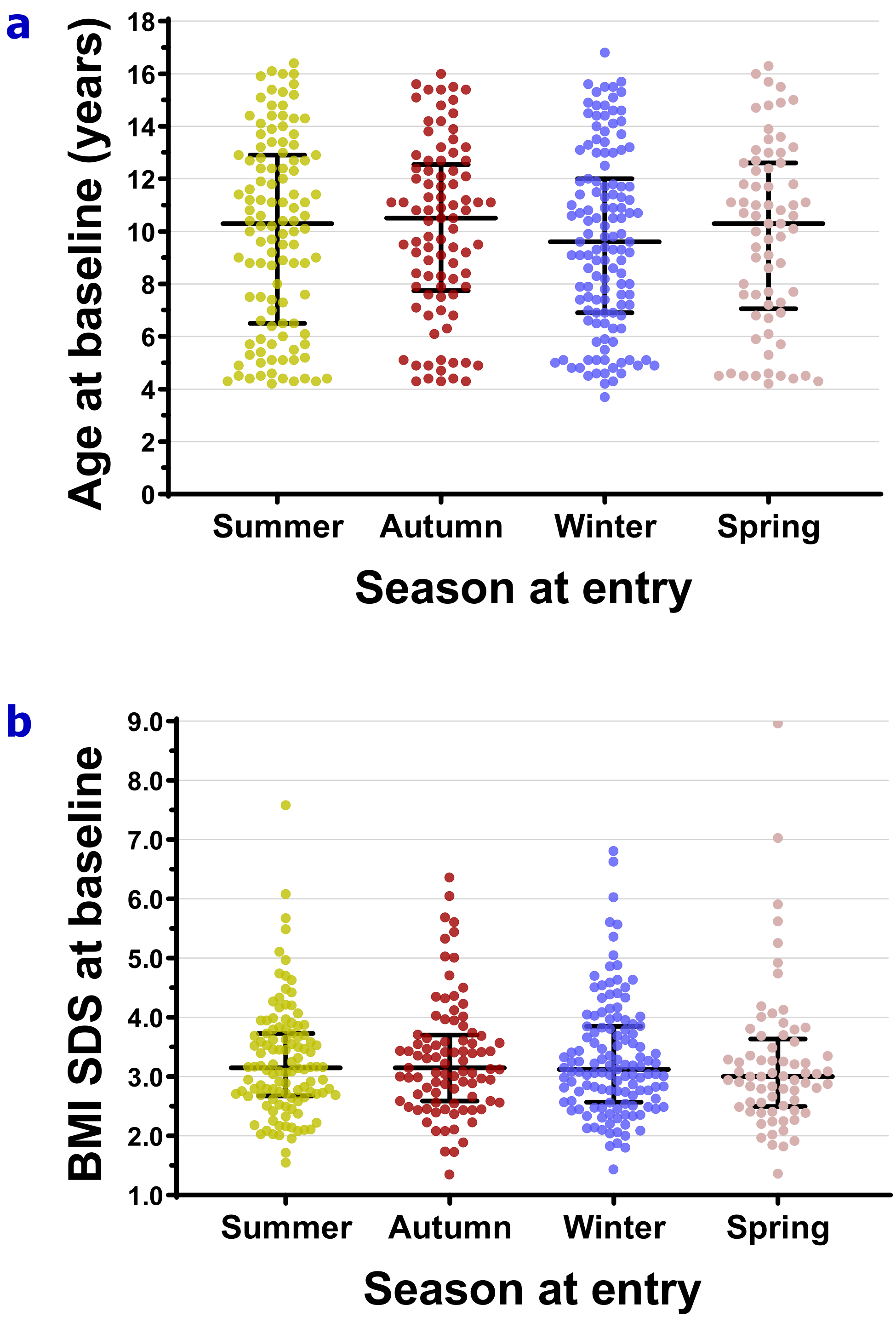

Supplementary Figure 3

Changes (Δ) in body mass index standard deviation scores (BMI SDS) at 6 months among Whānau Pakari participants in the randomised clinical trial (red) and service (blue) according to season at programme entry.

Data are presented as the adjusted means and 95% confidence intervals derived from a generalised linear mixed model adjusted for season at entry, programme cohort, a season*cohort interaction term, sex, ethnicity, age, and the BMI SDS value at baseline, as well as family ID as a random factor. *P<0.05, †P<0.01, ‡ P<0.001, and §P<0.0001 for within subgroup (cohort by season) differences compared to baseline. The colour-coded values above the *x*-axis are the proportions of participants in a given subgroup who had a BMI SDS reduction at 6 months. There were no statistically significant differences between cohorts among entrants in summer (P=0.85), autumn (P=0.37), winter (P=0.79), or spring (P=0.86).

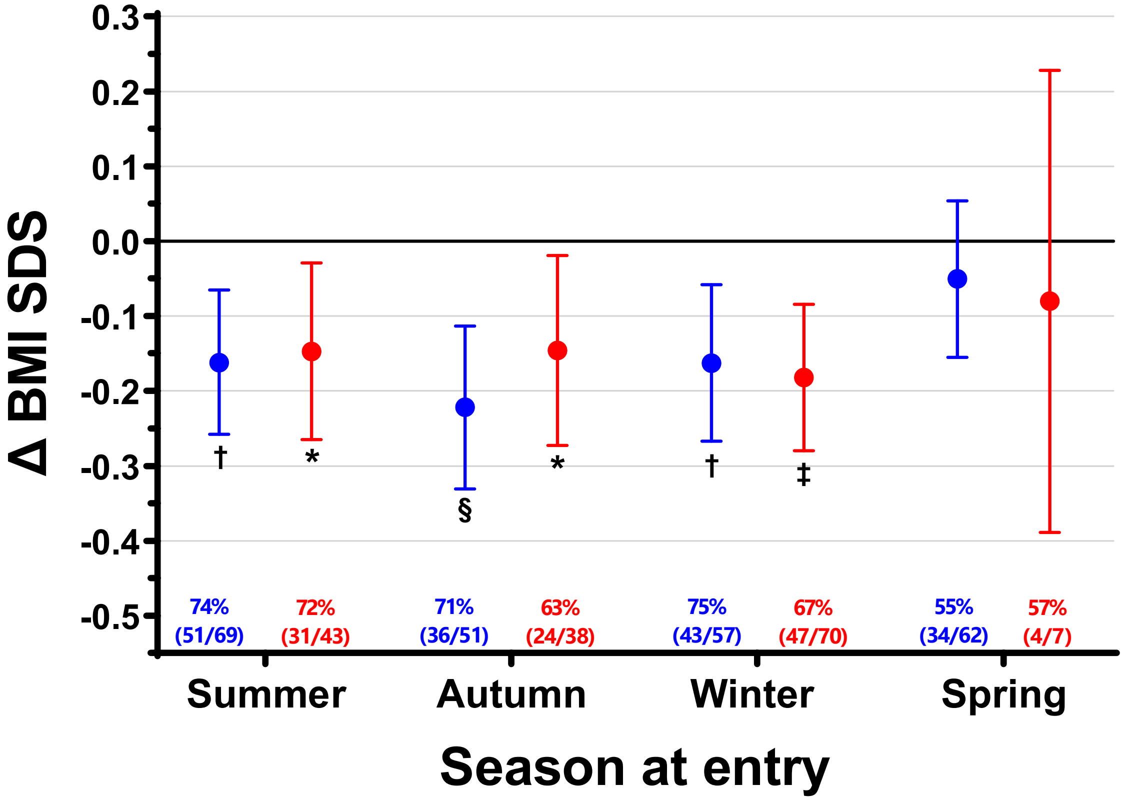

Supplementary Figure 4

Changes (Δ) in body mass index standard deviation scores (BMI SDS) based on the UK 1990 (UK Cole) reference, among participants in the Whānau Pakari programme approximately 6 months after entry.

a) Sinaplot showing Δ BMI SDS values for all study participants; the values provided are the proportions of participants who experienced a BMI SDS reduction at the 6-month follow-up.
b) The same data as in panel *a* but provided by season at entry.
c) The data displayed graphically and provided as values are the least-squares means and 95% confidence intervals (CIs) for each season derived from a generalised linear mixed model adjusted for baseline BMI SDS.
d) Adjusted means and 95% CIs for each season derived from generalised linear mixed models adjusted for season at entry, cohort (service or trial), sex, ethnicity, age, and baseline BMI SDS, as well as family ID as a random factor.

The horizontal bars indicate the lower quartile, median, and upper quartile of the distribution. ‡$P<0.001$ and §$P<0.0001$ for within-season differences in BMI SDS compared to baseline. There were no statistically significant differences between seasons from the analyses in panels C or D. Seasons were defined by the meteorological criteria (Trenberth KE, Bull Am Meteorol Soc 1983;64:1276–82); SDS were derived as per Cole et al. (Arch Dis Child 1995;73:25–9).

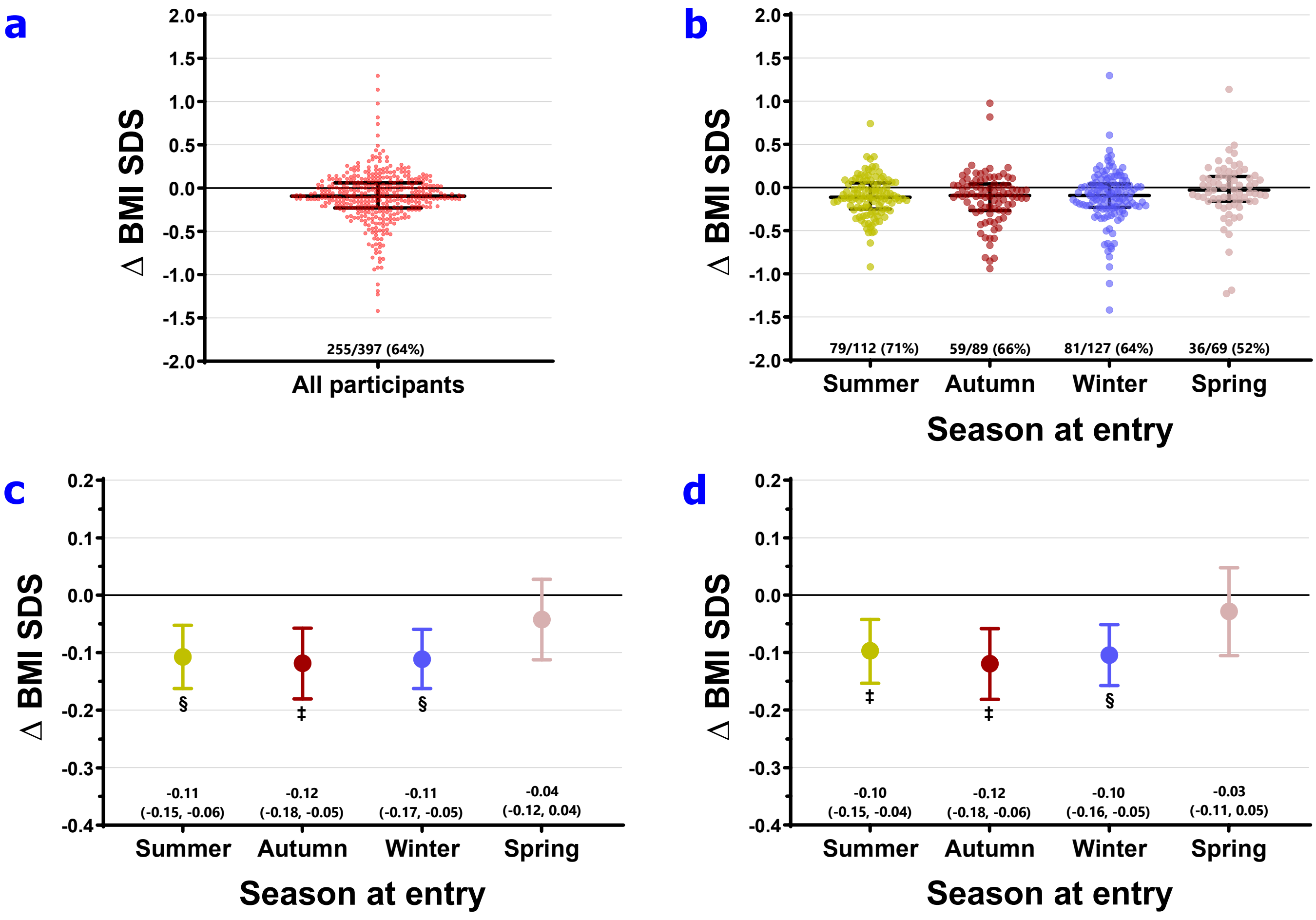

Supplementary Figure 5

Random-forest predicted 6-month change (Δ) in body mass index standard deviation score (BMI SDS) based on starting BMI SDS and age.

a) Baseline body mass index standard deviation (BMI SDS) vs model-predicted Δ BMI SDS at 6 months.
b) Baseline age vs model-predicted Δ BMI SDS at 6 months.
In both plots, each point represents one participant. The blue curve shows the smoothed trend in the data, with the shaded band representing its 95% confidence interval. Negative values on the *y*-axis indicate a predicted reduction (improvement) in BMI SDS. These predictions were generated by a random forest model using a robust cross-validation method (out-of-fold; OOF), ensuring the prediction for each person was made by a model that had not included their specific data. The plots show how each factor relates to the outcome on its own and do not imply causation.

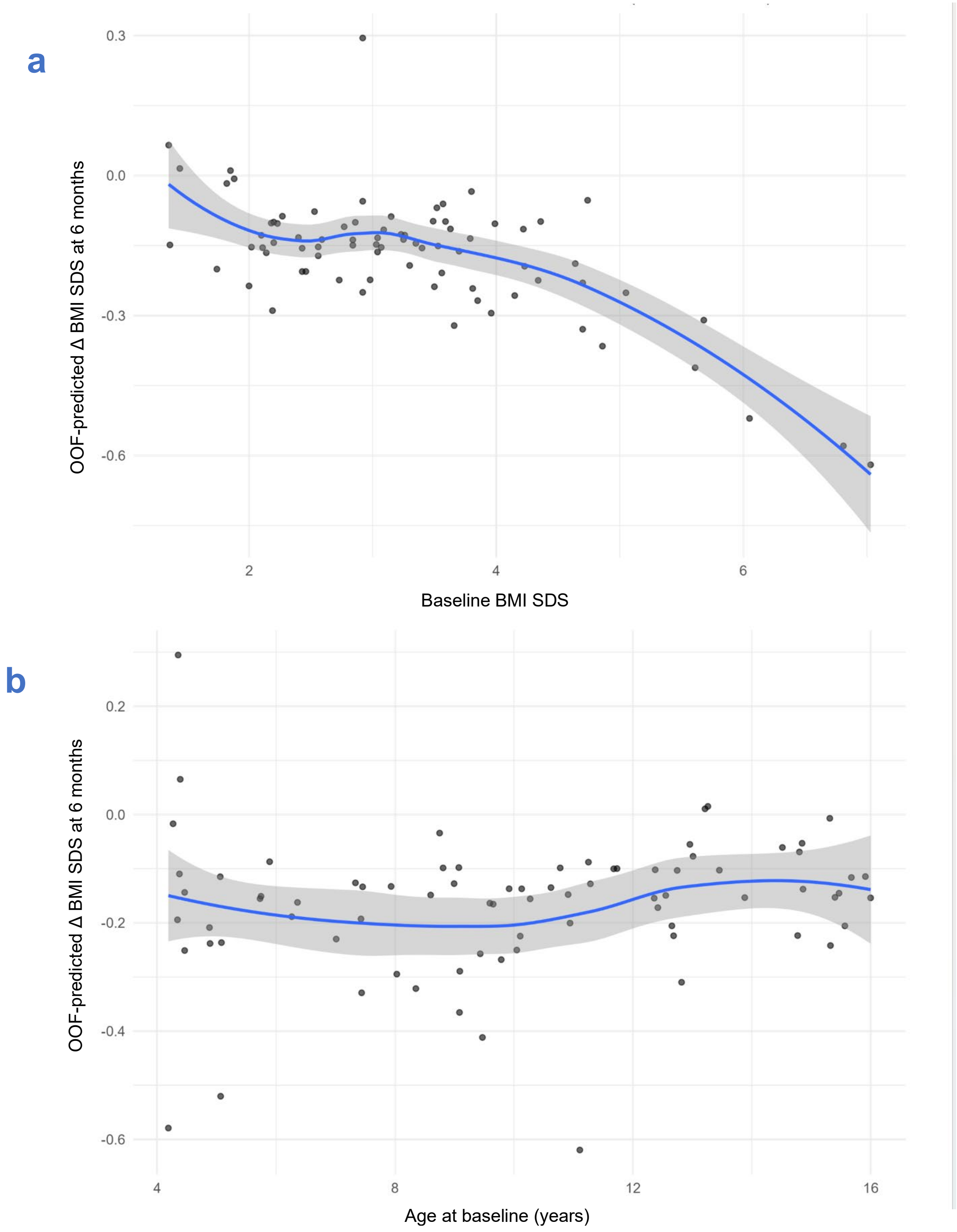

Supplementary Table 1

Baseline self-reported dietary habits and lifestyle characteristics among study participants overall and according to the season at Whānau Pakari programme entry.

DIETARY & LIFESTYLE FACTOR	SUMMER	AUTUMN	WINTER	SPRING	*P*
**Perceived physical activity level (minutes/day)**	112<br>76 [44, 116]	87<br>69 [35, 130]	126<br>68 [42, 99]	66<br>64 [39, 106]	0.59
**Time spent on screens (minutes/day)**	112<br>152 [76, 249]	87<br>154 [86, 283]	125<br>137 [97, 227]	67<br>154 [86, 236]	0.94
**Fruit/vegetable consumption (servings/day)**	110<br>2 [1, 3]	88<br>2 [1, 3]	127<br>2 [2, 3]	69<br>2 [1, 4]	0.63
**Intake of sweet drinks[1] (mL/day)**	111<br>179 [71, 500]	88<br>107 [42, 250]	126<br>143 [36, 250]	69<br>152 [36, 357]	0.56
**Sleep duration (hours/day)**	110<br>10.0 [9.5, 11.0]	87<br>10.0 [9.5, 11.0]	125<br>10.5 [10.0, 11.0]††† [2]	68<br>10.5 [10.0, 11.0]†	**0.023**

Data are the sample size for a given outcome (*n*), and the median [lower quartile, upper quartile].
The *P* values provided are for an overall difference between seasons and were derived from Kruskal-Wallis tests (highlighted in bold if statistically significant at $P<0.05$), with unadjusted pairwise comparisons between seasons examined using the Wilcoxon rank-sum tests.
†$P<0.05$ and †††$P<0.001$ for differences in comparison to autumn entrants;
[1] Sweet drinks: powdered drinks, cordial, fruit juice, energy drinks, and carbonated sweet drinks.
[2] $P=0.053$ compared to summer entrants.
Seasons were defined by the meteorological criteria (Trenberth KE, Bull Am Meteorol Soc 1983;64:1276–82).
